# Spatiotemporal analysis of diarrhea-related hospitalizations of children in Brazil’s Midwest region from 2011 to 2020

**DOI:** 10.1590/1980-549720240035

**Published:** 2024-06-24

**Authors:** Ana Lucia Sartori, Leila Regina de Oliveira, Maria Eduarda Pessatto

**Affiliations:** I Universidade Federal de Mato Grosso, Institute of Health Sciences – Sinop (MT), Brazil.

**Keywords:** Diarrhea, Child health, Spatial analysis, Epidemiological monitoring, Morbidity, Diarreia, Saúde da criança, Análise espacial, Monitoramento epidemiológico, Morbidade

## Abstract

**Objective:**

To examine spatiotemporal variability and identify clustering patterns of hospitalization rates for diarrhea in children younger than five years in Mato Grosso, Brazil, from 2011 to 2020.

**Methods:**

An ecological study was conducted using hospitalization records associated with diarrhea from the Brazilian Hospital Information System/Unified Health System. The relative risk of hospitalization for diarrhea in each municipality was calculated using SaTScan software considering a statistical significance level of 5% and 999 Monte Carlo replications.

**Results:**

A total of 13,315 diarrhea-associated hospitalizations for 5-year-old children were recorded. From 2011 to 2020, the annual rates for hospitalizations related to diarrhea decreased from 8.50 to 3.45/1,000 live births among children younger than one year and from 4.99 to 1.57 for children aged 1–4 years. Clusters of municipalities with high relative risk for hospitalizations due to diarrhea, statistically significant, predominated in the North, Northeast, and Southwest health administrative macro-regions of Mato Grosso for both age groups until 2016. From 2016 to 2020, clusters of the lowest relative risk were identified in the North and Center South health administrative macro-regions for children younger than five years.

**Conclusion:**

Results showed that hospitalization rates for diarrhea in children younger than five years reduced with the presence of low-risk clusters in Mato Grosso in the final years of the study. Public health surveillance should incorporate spatial analysis to investigate the diarrhea-related morbidity.

## RESUMO

**Objetivo:**

Examinar a variabilidade espaçotemporal e identificar *clusters* de hospitalização por diarreia em crianças menores de cinco anos de idade de Mato Grosso, de 2011 a 2020.

**Métodos:**

Um estudo ecológico foi conduzido utilizando os registros de hospitalização por diarreia do Sistema de Informação Hospitalar/Sistema Único de Saúde. O risco relativo de hospitalização por diarreia em cada município foi calculado utilizando o software SaTScan, considerando-se o nível de significância estatística de 5% e 999 replicações de Monte Carlo.

**Resultados:**

O total de 13.315 hospitalizações por diarreia em crianças menores de cinco anos de idade foram registradas. As taxas anuais de hospitalização por diarreia reduziram de 8,50 para 3,45/1.000 nascidos vivos em crianças menores de um ano de idade e de 4,99 para 1,57 em crianças de 1–4 anos de idade, de 2011 a 2020. *Clusters* de municípios com alto risco relativo para hospitalização por diarreia, estatisticamente significantes, predominaram nas macrorregiões administrativas de saúde Norte, Nordeste e Sudoeste de Mato Grosso para crianças de ambos os grupos etários, até 2016. De 2016 a 2020, *clusters* de baixo risco relativo foram identificados nas macrorregiões administrativas de saúde Norte e Centro-Sul para crianças menores de cinco anos de idade.

**Conclusão:**

Os resultados mostraram redução das taxas de hospitalização por diarreia em crianças menores de cinco anos de idade, com a presença de *clusters* de baixo risco em Mato Grosso nos últimos anos estudados. Os monitoramentos de saúde pública devem incorporar análises espaciais na investigação da morbidade por diarreia.

## INTRODUCTION

Diarrhea is a preventable disease that remains a key threat to the global health of children younger than five years^1,2^. In this age group, the disease affects nearly 1.7 billion children annually and kills 525,000 approximately^2^. In Brazil, the mortality rates due to diarrhea in children under five years are among the major causes of mortality^3,4^.

Several factors cause diarrheal diseases, including water contamination, poor sanitation facilities, unhygienic conditions, malnutrition, and infection^2,5,6^. Norovirus (22.2%) and Shigella (19.2%) are the leading etiologies in Central and South America, respectively. However, rotavirus remains the main etiologic agent related to moderate-to-severe diarrhea diseases in other regions of the world where the rotavirus vaccine has not been introduced^7^.

Diarrhea is defined as the passage of three or more loose or liquid stools per day. Dehydration is the major complication of severe diarrhea, and young children are more vulnerable than older children to dehydration. The treatment of dehydration using oral rehydration therapy is highly effective and can be conducted both at home and in outpatient facilities^8^. However, approximately 35.2% of children present moderate-to-severe episodes of diarrhea requiring hospitalization^9^.

Hospitalization for the disease in childhood causes a significant burden on households and health systems. The treatment of acute diarrhea in children in upper-middle-income countries that have not introduced the rotavirus vaccine is associated with direct medical costs ranging from US$ 12 to US$ 256 in primary health centers and tertiary public hospitals, respectively^10^. Using a modeling study, estimated direct medical costs accounted for 79% of the total direct costs^11^. The average cost of illness estimated for children is US$ 4.30 per outpatient episode and US$ 85.85 per inpatient episode. In addition to the economic impact, hospitalization for diarrhea affects the education of children younger than five years due to the loss of school days and caregiver workdays, which affect cognitive development and family resources, ultimately influencing health outcomes^12^. In Brazil, the rates of hospitalization among children of that age decreased by 52.5% from 2006 to 2018 and ranged from 68.4/10,000 to 32.5, respectively^4^. During the same period, Mato Grosso showed a reduction in hospitalization rate of 8.5% (annual percentage change; p<0.001); however, there is currently no knowledge about the homogeneity of this reduction for all municipalities of the state. Considering that the burden of diarrhea is associated with significant morbidity, from dehydration to childhood growth failure, extending beyond episodes progressing to death, surveillance of the disease remains necessary. We consider the application of spatial analysis, an essential tool, that can expand the understanding of the distribution of childhood diarrhea disease in Mato Grosso and contribute to the functioning of diarrhea surveillance^13-16^. Thus, this study aimed to examine spatiotemporal variability and identify clustering patterns in children younger than five years in a long-term time series in the state of Mato Grosso, Brazil.

## METHODS

### Design and study area

An ecological study was conducted using spatial scan statistics and administrative data of hospital admissions for diarrheal diseases in children younger than five years in the state of Mato Grosso from January 2011 to December 2020. The units of analysis were 141 municipalities in Mato Grosso.

**Figure 1.**
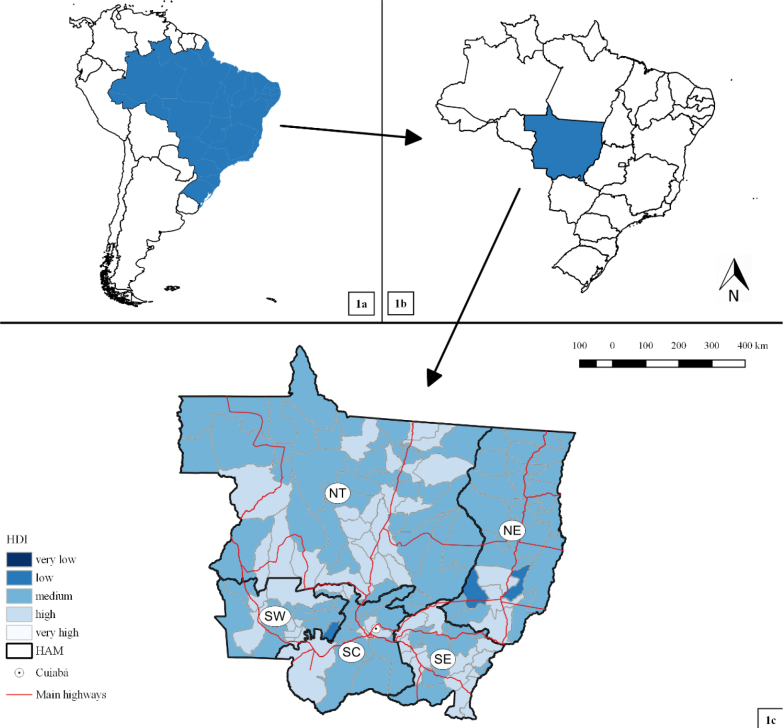
Geographic location of the study area and HDI

Mato Grosso is a state located in the center of the South American continent in the Midwest region of Brazil (Figures 1a and 1b). It is Brazil’s third largest state area (903.207,047 km²) and comprises the Legal Amazon^17,18^. It is divided into five health administrative macro-regions (HAMs): North, Northeast, South Center, Southeast, and Southwest (Figure 1c). In 2010, most municipalities achieved a medium (89/141, 63.1%) and high (49/141, 34.8%) Municipal Human Development Index (HDI) (https://www.undp.org/pt/brazil/idhm-munic%C3%ADpios-2010) (Figure 1c).

(a) Brazil in the South American region. (b) Mato Grosso state in Brazil. (c) The five-health administrative macro-regions (HAMs) of the state of Mato Grosso and respective Human Development Index (HDI) in 2010, using a scale of blue, where darker blue levels represent very low HDI, while light blue represents a high HDI. State’s capital; NT (North), NE (Northeast), SW (Southwest), SC (South Center), SE (Southeast). Light gray lines delimit the municipalities; red lines indicate main highways; black lines establish the HAMs of the state of Mato Grosso. HDI: very low 0–0.499; low 0.500–0.599; medium 0.600–0.699; high 0.700–0.799; very high 0.800–1.000.

In 2020, the estimated population in Mato Grosso was 3,455,092 inhabitants, comprising approximately 244,387 (7.0%) children younger than five years^19^. The infant mortality rate varied from 19.6/1,000 live births in 2010 to 15.4 in 2019^20^.

### Data source

Data were obtained from the Brazilian Hospital Information System/Unified Health System (*Sistema de Informação Hospitalar/Sistema Único de Saúde* [SIH-SUS]), which are available on the Department of Informatics of SUS (*Departamento de Informática do SUS* [Datasus]) portal without personal identification information (https://datasus.saude.gov.br/). As Datasus is in the public domain, approval from an Ethics Review Board was not required.

The SIH-SUS includes information on all hospital admissions that are funded by the SUS, Brazil’s publicly funded healthcare system. In Mato Grosso, in 2020, 78% of all hospitalizations of children younger than five years were recorded in the SIH-SUS^21^. The Hospital Admission Authorization (*Autorização de Internação Hospitalar* [AIH]) is the official document that constitutes each hospitalization record in the SIH-SUS. The AIH contains data that identify the patient and the services provided during hospitalization, including discharge diagnoses according to the International Classification of Diseases codes, 10th revision (ICD-10). The SIH-SUS variables of interest were the municipality of residence, date of birth, date of hospital admission, and discharge diagnosis (ICD-10 code). We excluded records with inaccurate or missing data for the following variables: municipality residence code, date of birth, and date of hospital admission. Age was calculated using the date of birth and hospital admission. Data were extracted in May 2022.

The outcome of interest was diarrhea-associated hospitalization with ICD-10 codes A08 and A09 as discharge diagnoses in SIH-SUS^4^.

For this investigation, the following two age groups were considered: < 1 year old and 1–4 years old. We addressed the rates for the age groups to compare the magnitude of the rates and evaluate whether the spatiotemporal distributions were similar. In the literature, children aged one year had higher rates of total diarrhea disability, hospitalization, and death than those aged 1–4 years^12,13^.

The population at risk was estimated for both age groups. The annual population at risk estimates for children younger than one year were defined as the number of live births recorded in the Brazilian Information System on Live Birth (*Sistema de Informação sobre Nascidos Vivos* [SINASC]) and available on the Datasus portal. For children aged 1–4 years, annual population estimates were obtained by interpolation based on data from the 2000 and 2010 Brazilian Census, National Institute of Geography and Statistics (*Instituto Brasileiro de Geografia e Estatística* [IBGE]; https://sidra.ibge.gov.br/home/pimpfbr/brasil). However, 15 of the 141 municipalities in Mato Grosso did not have data from the 2000 Brazilian Census, making a population estimate by interpolation unfeasible. For this reason, the Lexis diagram was applied to obtain the population at risk in the 15 municipalities. The Lexis diagram provides a way of representing the relationship between periods and cohorts, showing the number of those who survived from birth to the next projection period^22^.

### Data analysis

#### Descriptive analysis

The rates for diarrhea-associated hospitalizations were calculated by age group and year for the state and the municipalities using the number of hospitalizations as the numerator and children at risk as the denominator by age group and year, then multiplying the result by 1,000.

#### Spatiotemporal analysis

The SaTScan scanning technique following the discrete Poisson model was applied to explore clusters with a population that was susceptible or not to an event through spatial randomness tests^23^. Spatiotemporal analysis was adjusted to simultaneously scan for clusters with either high or low rates to derive the correct statistical inference. Elliptic window scanning was chosen using years as reference times (2011–2020). In this analysis, the window with maximum likelihood is defined as the most likely (primary) cluster not to have occurred by chance, followed by the secondary cluster; all presented with their respective periods, with their significances determined by the Monte Carlo simulation, with possibilities of 999 permutations in the randomization process and the attribution of a statistically 5% significant p-value^24^. A statistically significant cluster was defined using a maximum population size of 50% within the cluster and a maximum cluster size of 150,000 Cartesian units on the maximum axis of the cluster. The SaTScan software calculated the relative risk (RR) of each municipality based on the incidence of hospitalization for diarrhea. The RR was the estimated risk inside the cluster divided by the risk outside the cluster, defined by the following formula:

$$RR=\frac{c/E[c]}{\left(C\text{-}c)/(E[C]\text{-}E[c]\right)}=\frac{c/E[c]}{\left(C\text{-}c)/(C\text{-}E[c]\right)}$$

where *c* is the number of observed cases within the cluster, *C* is the total number of cases in the dataset, and *E[C* ^©^ *] = c* is the expected number of cases inside the window under the null hypothesis. When the sample size was small, the analysis was conditioned on the total number of cases observed^24^.

Thematic maps were constructed using QGis software (version 2.18) to visualize the distribution of the rates of diarrhea-associated hospitalization year-by-year for the age group and the RR clusters.

## RESULTS

**Figure 2.**
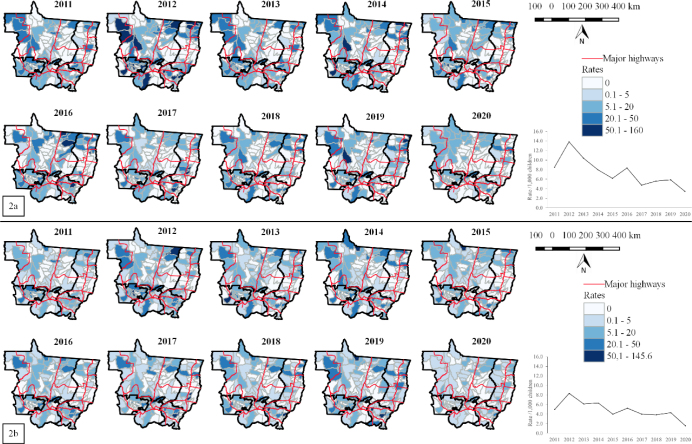
Trends of annual rates for diarrhea-associated hospitalizations for children younger than one year (a) and for children aged 1–4 years (b). Annual spatial variation maps of the rates at the municipality level in Mato Grosso and health administrative macro-regions, 2011–2020.

From January 2011 to December 2020, there were 13,315 diarrhea-associated hospitalizations recorded for children younger than five years. The number of cases was more frequent in children aged 1–4 years (70.4%), but children younger than one year presented higher incidence rates of hospitalization for diarrhea. From 2011 to 2020, the annual rates for diarrhea-associated hospitalizations in Mato Grosso progressively decreased from 8.50 to 3.45/1,000 live births among children younger than one year, and from 4.99 to 1.57 for children aged 1–4 years (Figures 2a and 2b and S1 Figure).

Light gray lines delimit the municipalities; red lines indicate main highways; and black lines establish the health administrative macro-regions of the state of Mato Grosso.

The thematic maps show the reduction in spatial variation of hospitalization rates for diarrhea at the municipality-level over the ten years of study. There was a punctual place during which the rate reduction was slow and others during which high rates remained stable for prolonged period (Figure 2).

**Figure 3.**
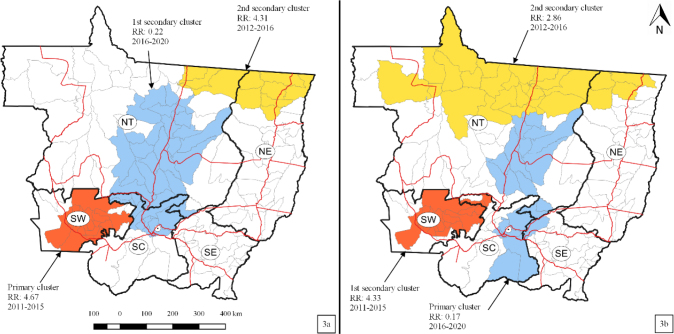
Spatiotemporal clusters (high and low rates) of hospitalizations for diarrhea among children younger than one year (a) and for children aged 1–4 years (b) in Mato Grosso, 2011–2020.

Figure 3a displays the three significant clusters of municipalities with hospitalizations for diarrhea in children younger than one year in Mato Grosso, and Table 1 shows two of them with a statistically significant high RR. The primary cluster was in the Southwest HAM of Mato Grosso (RR 4.67; p<0.0001), comprising 17 municipalities and 647 cases recorded from 2011 to 2015. A first secondary cluster was identified in the North and South Center HAMs of the state (RR 0.22; p<0.0001) from 2016 to 2020, accounting for 34 municipalities and 256 hospitalization records. The second secondary cluster identified (RR 4.31; p<0.0001) occurred from 2012 to 2016 in ten municipalities from the North and Northeast HAMs of the state, with 353 hospitalization records.

Picture a: Orange color indicates the primary cluster (RR=4.67); blue indicates the first secondary cluster (RR=0.22); and yellow indicates the second secondary cluster (RR=4.31). Picture b: Blue color indicates the primary cluster (RR=0.17); orange indicates the first secondary cluster (RR=4.33); and yellow indicates the second secondary cluster (RR=2.86).State’s capital; NT (North), NE (Northeast), SW (Southwest), SC (South Center), and SE (Southeast). Light gray lines delimit the municipalities; red lines indicate the main highways; and black lines establish the health administrative macro-regions of the state of Mato Grosso.

**Table 1:** Spatiotemporal clusters of children younger than five years with diarrhea in Mato Grosso, 2011–2020.

Age group and clusters	N	Time frame	Observed*	Expected^†^	RR	LLR	p-value
<1 year							
Primary	17	January 1, 2011 to December 31, 2015	647	159.15	4.67	452.46	<0.001
First secondary	34	January 1, 2016 to December 31, 2020	256	939.42	0.22	423.04	<0.001
Second secondary	10	January 1, 2012 to December 31, 2016	353	87.99	4.31	234.73	<0.001
1–4 years							
Primary	18	January 1, 2016 to December 31, 2020	402	1,910.90	0.17	1,025.45	<0.001
First secondary	18	January 1, 2011 to December 31, 2015	1,582	420.17	4.33	1,014.44	<0.001
Second secondary	26	January 1, 2012 to December 31, 2016	1,532	600.08	2.86	555.33	<0.001

N: number of municipalities; RR: relative risk; LLR: log likelihood ratio.

* Number of observed cases in a cluster;

† Number of expected cases in a cluster.

As displayed in Figure 3b and Table 1, the primary cluster (RR 0.17; p<0.0001) for children aged 1–4 years accounted for 402 cases distributed in 17 municipalities in the North and South Center HAMs and in one municipality from the Southeast, from 2016 to 2020. The first secondary cluster presented the statistically significant highest RR (RR 4.33; p<0.0001); it involved 15 municipalities from the Southwest and three from South Center HAMs from 2011 to 2015 and consisted of 1,582 cases. The second secondary cluster (RR 2.86; p<0.0001) included the North and Northeast HAMs of Mato Grosso (1,532 cases) from 2012 to 2016 (Figure 3b and Table 1).

## DISCUSSION

This study highlights the spatiotemporal dynamics of hospitalization for childhood diarrhea in a state of Brazil’s Legal Amazon from 2011 to 2020. This is the first study to produce health evidence on the spatiotemporal risk of diarrhea-associated hospitalization in children younger than five years in Mato Grosso.

We found higher rates of hospitalization for diarrhea in children younger than one year, similar to those found in the state of Tocantins, Brazil^13^. Children younger than one year may be more affected by diarrheal infections/diseases during childhood, possibly because of factors such as non-adherence to exclusive breastfeeding in the first six months of life, mothers’ lack of education, and difficulty accessing health services^25,26^.

We observed a reduction in hospitalization rates for diarrhea in children younger than five years in Mato Grosso during the study period. As diarrhea is a multifactorial disease^2,5,6^, several interventions implemented over time might have contributed to the decline in the rates and elimination of high-risk clusters in the HAMs Southwest, North, and Northeast of the state. For these HAMs, the years 2015 and 2016 temporarily delimited the elimination of high-risk clusters.

Diarrhea (gastroenteritis and complications) is a cause of hospitalization for primary care-sensitive conditions (PCSCs), which is used as a proxy for access and quality of health services in primary health care (PHC)^27^. In the SUS network, PHC constitutes the gateway for childcare, before the need for hospitalization. In Mato Grosso, there was an increase in the coverage rates of PHC units, from 69.88% to 79.47%, from January 2001 to December 2020 (E-gestor)^28^. In the municipalities included in the high- and low-risk clusters of hospitalization for diarrhea in children, an upward trend was identified in the mean coverage rates of PHC units over the study period (S1 Table). The increase in Family Health Strategy coverage and the expansion of the National Program for Access and Quality Improvement in PHC were associated with reduced hospitalization for childhood diarrhea in Brazil^29-32^. In PHC, PCSCs have a greater chance of being followed up at an outpatient level without the need for hospitalization, minimizing the chance of increasing the severity as gastroenteritis is detected early and treated effectively^16^.

Lack of adequate sanitation, untreated drinking water, neighborhood infrastructure, household conditions, and poverty are factors directly related to childhood diarrhea^5,6^. Among other factors, such as nutrition and hygiene behavior^5,6^. The National Conditional Cash Transfer Program (*Bolsa Família*) supported an expressive number of families, acting as an increment factor for the acquisition of consumer goods or improvement of food quality. The combined conditional cash transfer program and environmental health interventions on diarrhea morbidity in children in Brazil resulted in a positive effect^31,33^. Additionally, the spread of rotavirus vaccine use in childhood immunization programs since 2006 has contributed to a reduction in the morbidity and mortality rates of diarrheal diseases in children worldwide and Brazil, including the state of Mato Grosso^4,12,34-36^. The proportion of hospitalizations attributable to rotavirus was 50% lower than that in countries that had introduced the rotavirus vaccine, compared to those that had not introduced it^7^.

Since 2016, low-risk clusters have been identified for children of both age groups. Low-risk clusters are concentrated in the South Center HAM of the state and include the capital, Cuiabá. Most municipalities in these clusters are located around a major federal highway, BR-163, and inside the Cerrado biome. Much of the economic development and population growth in the state of Mato Grosso occurred along the banks of BR-163. Moreover, these clusters contain the populated municipalities and those with the highest HDI compared to the other clusters identified in the study (Figure 1). HDI is an important indicator of relevant measures for diarrheal disease among children^37^. The data from the Global Analysis and Assessment of Sanitation and Drinking-Water by the World Health Organization revealed a correlation of high HDI with the use of improved drinking water sources and improved sanitation facilities in 75 countries, including Brazil^37^. Mato Grosso ranked 11th among the highest HDI in Brazil^38^. For this reason, the better conditions for preventing diarrhea reflected by the higher HDIs have probably contributed to low-risk clusters of hospitalization for the disease. This contrasts with the setting identified in Alagoas state (which has the worst HDI)^39^ for mortality rates of diarrhea for both age groups from 2011 to 2020 (S1 Figure).

Although the rates have reduced in the state, there remain municipalities with considerable rates of hospitalization for diarrhea that should not be neglected, with higher rates than those described for other regions of the country^4^. These municipalities are located, mainly, in the Southwest, Northeast, and North HAMs (data not shown). Southwest HAM is considered a medium socioeconomic development area and still has a low availability of health services; most of the municipalities originated from gold and diamond mining^40,41^. The Northeast and North HAM are bathed by the Amazon basin with areas inhabited by several indigenous ethnic groups. Both HAMs are considered of low socioeconomic development, low availability of health services, and low population density^40,41^.

Mato Grosso has an extensive territorial area and is in full development. Temperature and soil moisture precipitation can also influence the survival time of enteropathogens outside their hosts. For most etiologic agents, elevated temperature and humidity can impact diarrhea rates^42^. Considering that Mato Grosso comprises three biomes, including the Amazon, Cerrado, and Pantanal^43^, this diversity should not be disregarded in future studies on hospitalizations for diarrhea in the municipalities of the state.

A reduction in viral and non-viral infections was documented in Brazil during the first year of the COVID-19 pandemic^44-46^. In France, a time-series study from 2017 to 2020, reported a decrease of more than 70% in acute gastroenteritis, common cold, and acute otitis media pediatric emergency visits, which was associated with lockdown and school closures due to the COVID-19 pandemic^46^. We observed a decrease in hospitalization rates for diarrhea in 2020 for both age groups, compared to the other years of the study. However, this result and its relationship with the COVID-19 pandemic should be interpreted with caution, as the rates showed a statistically significant downward trend before 2020 (S2 Table).

This study has potential limitations. First, the SIH covers only SUS hospitalizations. However, the records used in our study included approximately 78% of hospitalizations in children younger than five years living in Mato Grosso, representing a considerable portion of the pediatric population^21^. Additionally, the pathogens of cases of diarrhea were not identified in the medical records, and there were possible discharge diagnostic errors. Moreover, the anonymous records used made it impossible to classify the first hospitalization record for diarrhea and recurrent cases.

In summary, our study identified a reduction in hospitalization rates for diarrhea in children younger than five years of age based on the existence of clustering patterns in different periods and HAMs in the state of Mato Grosso, where some of these represented high-risk clusters. Low-risk clusters for hospitalization rates of diarrhea among children younger than five years were identified from 2016 to 2020 and appear to be composed of municipalities with higher HDI. The findings reinforce the idea that routine public health surveillance should incorporate spatial analysis to investigate diarrheal morbidity. Spatiotemporal analysis may represent the cartography of underdevelopment and poverty, characterized mainly by the occurrence of preventable and curable diseases^47^. In this way, the evidence will serve to outline priority areas for health professionals, managers, and organizations to establish more effective and punctual actions for health promotion and prevention of diarrhea in childhood in Mato Grosso.
