# Supplementary material for: Spatiotemporal analysis of diarrhea-related hospitalizations of children in Brazil’s Midwest region from 2011 to 2020
Source: Rev Bras Epidemiol. 2024 Jun 24;27:e240035. doi: 10.1590/1980-549720240035 (PMC11207105; doi:10.1590/1980-549720240035)
Supplement: Supplementary file 1 [file 1980-5497-rbepid-27-e240035-suppl1.pdf]

S1 Table. Trends in average percentage of primary care coverage by cluster and age group in the state of Mato Grosso, 2011-2020. We selected only the municipalities of the state of Mato Grosso identified in the study clusters, by age group. The calculation of the average percentage considered the amount of each municipality's proportion of primary care coverage by months of January and December of the 2011-2020 period. Data were available in the public domain e-Gestor (https://egestorab.saude.gov.br/paginas/acessoPublico/relatorios/relHistoricoCoberturaAB.xhtml). We used Prais-Winsten generalized linear regression to analyze the trends in the average percentage of primary care coverage.

Age group and cluster	n	Time frame	Coefficient	CI 95%	p-value
**<1 year**					
Primary	17	Jan 1st 2011 to Dec 31st 2015	0.006	0.0040 to 0.0071	0.000
1st Secondary	34	Jan 1st 2016 to Dec 31st 2020	0.004	0.0025 to 0.0054	0.000
2nd Secondary	10	Jan 1st 2012 to Dec 31st 2016	0.001	-0.0014 to 0.0025	0.583
**1-to-4-years**					
Primary	18	Jan 1st 2016 to Dec 31st 2020	0.001	0.0003 to 0.0021	0.009
1st Secondary	18	Jan 1st 2011 to Dec 31st 2015	0.002	-0.0001 to 0.0037	0.056
2nd Secondary	26	Jan 1st 2012 to Dec 31st 2016	0.000	-0.0012 to 0.0021	0.566

S2 Table. Segmented regression for the hospitalization rates for diarrhea by age group in the state of Mato Grosso, 2011-2020. Joinpoint regression analysis was conducted in Joinpoint Trend Analysis Software Version 5.0.2 (https://surveillance.cancer.gov/joinpoint/) to detect changes in trends (segments) of the hospitalization rates for diarrhea by age-group during 2011-2020, in the state of Mato Grosso. Joinpoint regression analysis were conducted according to the presence or absence of the homoscedasticity in the hospitalization rates applied White's test (p<0.05). We detected the presence of the heteroscedasticity for the hospitalization rates for children 1 to 4 years (p=0.0230)** but not for the for children <1 year (p= 0.0778)*.

Segments	Test Statistic (t)	p
**<1 year***		
2011-2013	-0.2	0.864
2013-2020	-4.9	0.004
**1 to 4 years****		
2011-2013	0.0	0.969
2013-2020	-4.0	0.010

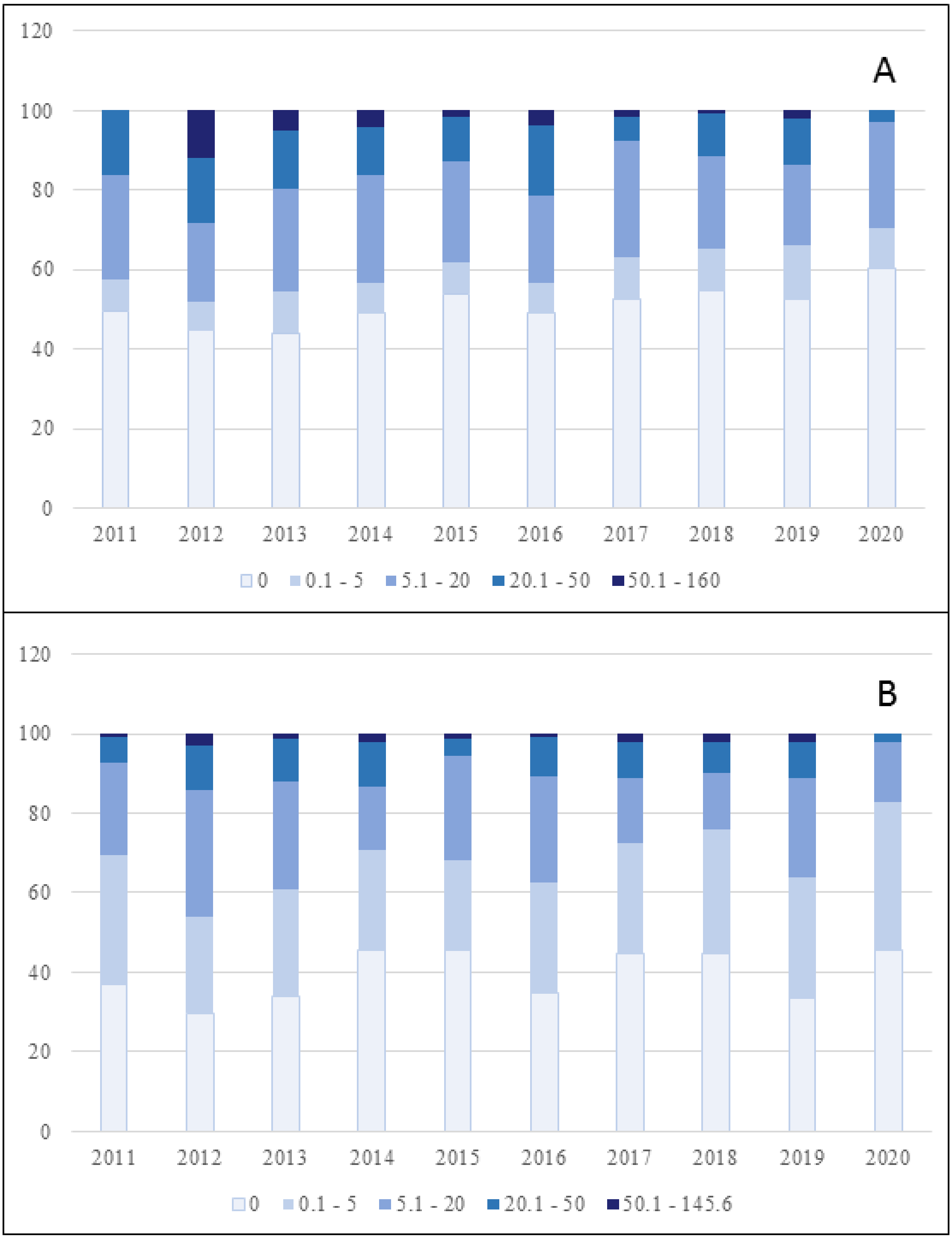

S1 Figure. Percentage of the municipalities according to the annual rates of hospitalizations for diarrhea among children under one year old (A) and for children aged 1 to 4 years old (B) in Mato Grosso, 2011-2020.
